# An Acetylenic Alkaloid from the Calcareous Sponge *Leucetta* sp.

**DOI:** 10.3390/md9030382

**Published:** 2011-03-21

**Authors:** Idam Hermawan, Nicole J. de Voogd, Junichi Tanaka

**Affiliations:** 1 Department of Chemistry, Biology and Marine Science, University of the Ryukyus, Nishihara, Okinawa 903-0213, Japan; E-Mail: damz_98@yahoo.com; 2 Netherlands Centre for Biodiversity, Naturalis, PO Box 9517, 2300 RA Leiden, The Netherlands; E-Mail: Nicole.devoogd@ncbnaturalis.nl

**Keywords:** sponge, acetylene, alkaloid, cytotoxicity

## Abstract

A new acetylenic alkaloid was isolated from the sponge *Leucetta* sp. The structure was established by analyzing spectroscopic data. The alkaloid showed cytotoxicity IC_50_ 2.5 μg/mL against NBT-T2 cells.

## 1. Introduction

More than 8000 species of sponges have been recorded from intertidal to deep oceanic floors, from tropical to polar regions, and from marine to freshwater environments [1]. The majority of sponges prefer to grow in shallow warm waters such as coral reefs. Since coral reef sponges have been major subjects from an early stage of marine natural products study, they are recognized as the most prolific sources of diverse bioactive secondary metabolites among macrobenthos [2]. However, dereplication has become an increasingly major issue in natural product chemistry as it is common to encounter known molecules probably reflecting the fact that many overlapping specimens have been examined. To overcome this problem and to increase the diversity of source organisms, we collected sponges in a coral reef twilight zone (50–100 m), where the depth is too deep for conventional scuba diving and the fauna there have not been well examined. A few examples from the zone include seragamides [3] and a unique fatty acid [4]. In this short note, we describe a structure of a new cytotoxic acetylenic alkaloid from a sponge collected in the zone.

## 2. Results and Discussion

**Figure 1.**
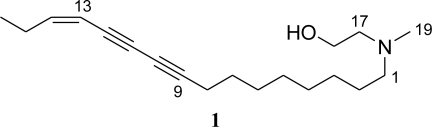
Structure of compound **1**.

The small sponge was extracted with acetone and its lipophilic portion showing cytotoxicity at 5 μg/mL was subjected to chromatographic separation to give compound **1** (0.088% from sponge). Compound **1** was found to have a molecular formula as C_19_H_31_NO indicating five degrees of unsaturation, which can be explained by the presence of two substituted acetylenes (δ_C_ 65.1 s, 71.9 s, 78.1 s, 84.8 s; 2334 cm^−1^) and one *cis* double bond (δ_H_ 5.44 brd, *J* = 10.8 Hz, 6.04 dt, *J* = 10.8, 7.5 Hz; δ_C_ 107.5 d, 149.2 d). Other structural features include an *N*-methyl group (δ_H_ 2.24 s (3H); δ_C_ 41.5 q), a terminal ethyl group (δ_H_ 1.01 t (3H), 2.36 m (2H)), a primary alcohol (δ_H_ 3.58 t (2H); δ_C_ 58.2 t; 3407 cm^−1^), and nine methylenes (δ_H_ 1.30–2.32, 2.39 m (2H), 2.52 t (2H)). The primary alcohol group was coupled (*J* = 6.7 Hz) to a methylene at δ_H_ 2.52, which showed HMBC correlation with the *N*-methyl group at δ_H_ 2.24 and also with another methylene at δ_H_ 2.39 indicating the presence of a tertiary amine with these substituents. The terminal ethyl group was connected to the double bond by observing COSY (H-13/H-14,15, H-14/H-15) and HMBC (H-13/C-15, H-15/C-13,14,16) cross peaks. This double bond was found to be conjugated to a diyne group connected to methylenes (H-13/C-11, H-14/C-12, H-8/C-9,10,11,12). By elucidating the remaining methylenes as a linear structural unit with HMBC (H-7/C-6,8,9, H-1/C-2,3, H-2/C-3), the whole structure was assigned as 2-(hexadec-13-ene-9,11-diynyl-methyl-amino)-ethanol (Figure 1).

A number of polyacetylenic molecules have been reported from marine sources [4–7], however, compound **1** is the first example of polyacetylene with an alkaloidal functionality from a marine sponge.

## 3. Experimental Section

### 3.1. General Procedures

FTIR spectrum was taken on a Varian FTS-3000 instrument. ^1^H, ^13^C and 2D (COSY, HSQC, HMBC) NMR spectra were obtained on a Bruker Avance III 500 spectrometer in CDCl_3_ with reference to an internal standard of TMS. Chemical shifts and coupling constants were given as δ and Hz. ESIMS was measured on a Jeol JMS-T100LP instrument.

### 3.2. Animal Material

**Figure 2.**
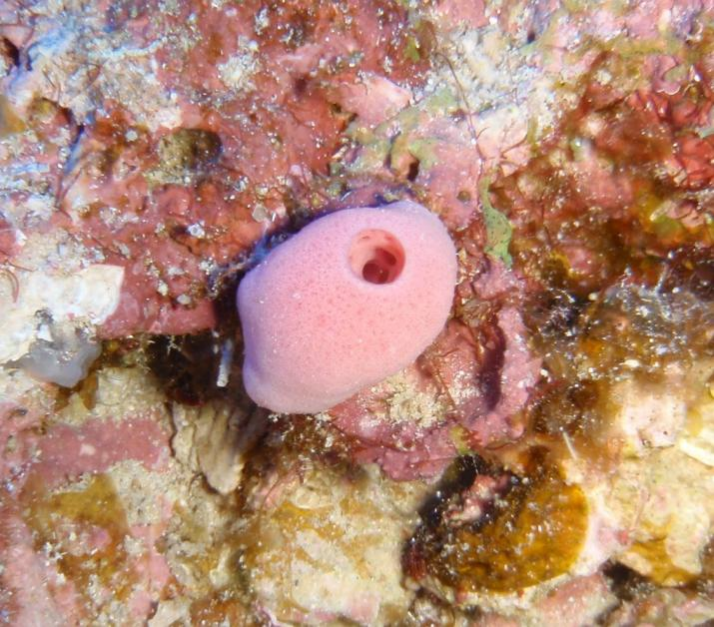
The sponge *Leucetta* sp.

The sponge, an undescribed *Leucetta* sp. (Figure 2, Leucettidae, Clathrinida, Calcarea), was collected at 50 m depth off Kume Island in Okinawa on September, 2009 and kept frozen until extraction. The sponge was identified by one of us (NJdV) and deposited at NCB Naturalis under the code RMNH POR 3927. The sponge is small, pink, pyriform and has one prominent osculum. The skeleton consists of regular triactines and tetractines densely and irregularly scattered throughout the ectosome and choanosome. The rays of the spicules range from 5–110 μm, of which the smaller ones are juvenile forms.

### 3.3. Isolation of Alkaloid **1**

The sponge (wet, 3.54 g) was extracted two times with acetone (30 mL). The resulting residue (0.31 g) was partitioned between EtOAc and water, and the organic layer was concentrated to give 14.7 mg of the extract. It was then separated on a silica gel column with stepwise elution using *n*-hexane-EtOAc (2–1, 1–1, and 1–2), EtOAc-MeOH (1–5), and MeOH to give a total of 7 fractions. Fraction 7 contained 3.1 mg (0.087%) of the alkaloid **1**.

### 3.4. Alkaloid **1**

Yellow oil, FTIR 3407, 2931, 2857, 2334, 1458, 1043 cm^−1^. ^1^H and ^13^C NMR: see Table 1. ESIMS obsd *m/z* 290.24798, calcd for C_19_H_32_NO^+^ 290.24784.

### 3.5. Cytotoxicity Testing

NBT-T2 cells (BRC-1370, purchased from Riken BioResource Center) were cultured in DMEM supplemented with 10% heat-inactivated fetal bovine serum and antimicrobials under standard protocol and seeded in 200 μL wells. After preincubation (37 °C, 24 h), cells were exposed to graded concentrations of compound **1** in duplicate (37 °C, 48 h). Then, the cells were treated with MTT solution (15 μL, 5 mg/mL in PBS) after removal of the medium and incubated for 3 h. The residual formozan was dissolved in DMSO (100 μL) and the absorbance was measured with a Tecan sunrise microplate reader at 560 nm. The IC_50_ values were found by plotting the absorbance values against concentrations. The alkaloid showed cytotoxicity IC_50_ 2.5 μg/mL against NBT-T2 cells.

## 4. Conclusions

A new acetylenic alkaloid **1** was characterized with spectroscopic methods. Together with our previous work [4], Calcareous sponges are still promising sources of unique bioactive molecules.

## Figures and Tables

**Table 1.** ^1^H and ^13^C NMR data for compound **1** in CDCl_3_.

**C#**	**δ_C_^a^**	**δ_H_ (mult., *J* in Hz)**	**COSY**	**HMBC**
1	57.7 t	2.39 m	H-2	C-2,3,17,19
2	27.2 t	1.46 m	H-1,3	C-3
3	27.2 t	1.30 m	H-2	C-1
4	29.3 t	1.30 m		
5	29.0 t	1.30 m		
6	28.8 t	1.39 m		C-5
7	28.2 t	1.53 m	H-6,8	C-6,8,9
8	19.6 t	2.32 m	H-7	C-9,10,11,12
9	84.8 s	-		
10	65.1 s	-		
11	78.1 s	-		
12	71.9 s	-		
13	107.5 d	5.44 brd, *J* = 10.8 Hz	H-14,15	C-11,15
14	149.2 d	6.04 dt, *J* = 10.8, 7.5 Hz	H-13,15	C-10
15	24.1 t	2.36 m	H-13,14,16	C-13,14,16
16	13.3 q	1.01 t, *J* = 7.6 Hz	H-15	C-14,15
17	58.7 t	2.52 t, *J* = 6.7 Hz	H-18	C-1
18	58.2 t	3.58 t, *J* = 6.7 Hz	H-17	C-17
19	41.5 q	2.24 s		C-17

a Multiplicities were determined by DEPT experiments.
